# Characterization of gut microbiome dysbiosis in calcium oxalate stone patients with comorbid metabolic syndrome

**DOI:** 10.3389/fmicb.2025.1644416

**Published:** 2025-11-25

**Authors:** Maimaitiaili Batur, Xun Li, Bide Liu, Shuheng Wang, Qiang Dong, Nueraili Abudurexiti, Zewei Liu

**Affiliations:** People's Hospital of Xinjiang Uygur Autonomous Region, Urumqi, China

**Keywords:** calcium oxalate stone, metabolic syndrome, gut microbiome, renal calculi, etiology

## Abstract

**Objective:**

Metabolic syndrome is an important risk factor for calcium oxalate stone, yet the underlying mechanism remain unclear. Gut microbiota is involved in human metabolic processes and is associated with both metabolic syndrome and calcium oxalate stone formation.

**Methods:**

In this study, 100 subjects were divided into four groups: calcium oxalate stone with metabolic syndrome (Group A), metabolic syndrome only (Group B), calcium oxalate stone only (Group C), and healthy controls (Group D), with 25 cases in each group. Gut microbiota composition and function were analyzed using *16S rRNA* gene sequencing. Microbiota diversity, species differences, and metabolic function changes were assessed by combining clinical parameters and metabolic pathway (KEGG) annotation.

**Results:**

The α diversity in Group A was significantly lower than in the other three groups (Shannon index, *P* < 0.05), and β diversity analysis revealed significant differences in bacterial community structure among all four groups (ANOSIM, *P* < 0.05). In Group A, short-chain fatty acid (SCFA)-producing probiotics (e.g., *Faecalibacterium, Faecalibacillus, Prevotella*) were reduced, while pro-inflammatory bacteria (e.g., *Eggerthella* and *Anaerobacteriaceae*) were enriched. RDA correlation analysis indicated that *Faecalibacterium* is negatively correlated with blood glucose levels, *Faecalibacterium* and *Roseburia* are positively correlated with urinary pH. KEGG analysis showed that the bisphenol degradation pathway was reduced (logFC = −1.45, *P* = 0.027) and the retinol metabolism pathway was enriched (logFC = 0.928, *P* = 0.006) in Group A compared to Group B.

**Conclusion:**

Patients with calcium oxalate stone and metabolic syndrome exhibit a “double imbalance” in gut microbiota: on the one hand, the reduced diversity of the microbiota and the decrease of SCFAs-producing microbiota weakened the metabolic protective effect of the gut microbiota; on the other hand, the enrichment of pro-inflammatory and pathogenic bacteria exacerbated metabolic disorders and inflammatory reactions. The present study reveals that gut microbiota play a role in the mechanism of metabolic syndrome promoting calcium oxalate stone formation, and these findings provide a theoretical basis for the use of probiotics to prevent calcium oxalate stone.

## Introduction

The prevalence and recurrence rate of urinary stone are extremely high, with a prevalence rate of about 5.6%−11.1% in China, and the incidence rate is increasing year by year. Most patients need repeated surgery, causing serious health and economic burden (Tan et al., 2023). The etiologic mechanism of primary calcium oxalate stone is still unclear, and their formation is regulated by multiple factors such as genetics, metabolism and environment (Shastri et al., 2023). Currently, calcium oxalate stone is considered to be a systemic metabolic disease, and Metabolic syndrome (MetS) is closely associated with it (Liu et al., 2024). The clustering of MetS traits is associated with greater kidney stone disease severity, with patients exhibiting all four characteristics facing an 1.8 fold higher risk of recurrent or multiple stones than those without MetS (Kohjimoto et al., 2013). However, there is no clear mechanism to explain the promotion of stone by MetS. Gut microbiota are involved in stone formation through oxalate metabolism and immune regulation. *Oxalobacter formigenes* (Oxf) degrades oxalate using key enzymes such as formyl-CoA transferase and oxalyl-CoA decarboxylase, but it is not the only stone - related bacterial species (Ticinesi et al., 2019). Patients with calcium oxalate stone exhibit abnormal microbiota diversity. Compared with healthy individuals, the relative abundances of harmful bacteria such as the genus *Bacteroides* and the genus *Escherichia - Shigella* increase, while those of probiotics such as *Lactobacillus, Bifidobacterium, Prevotella*, and *Faecalibacterium* decrease. These microbiota affect the progression of kidney stone through mechanisms such as oxalate metabolism, lipid regulation, and inflammatory responses (Yuan et al., 2023; Millán Rodríguez et al., 2020). Patients with MetS also exhibit reduced diversity and significant structural alterations in their gut microbiota. This dysbiosis impacts host metabolism, immunity, and inflammation, creating a vicious “microbiota-metabolism” cycle (Nie et al., 2024). Gut microbiota is involved in the development of stone and metabolic syndrome by influencing host metabolic status, immune function and inflammation, and metabolic syndrome is also closely related to stone and gut microbiota disorders. Based on this relationship, we analyzed the gut microbiota characteristics of calcium oxalate stone patients with metabolic syndrome by 16S rRNA sequencing, exploring the metabolic syndrome promoting oxalate calcium stone potential mechanisms.

## Materials and methods

### Study subjects

The study was approved by the Ethics Committee of Xinjiang Uygur Autonomous Region People's Hospital (Ethics No. KY20240130157). A total of 100 research subjects were enrolled in this study from February 2024 to December 2024, and all subjects signed an informed consent form. They were divided into four groups, calcium oxalate stone combined with metabolic syndrome group (Group A), metabolic syndrome alone group (Group B), calcium oxalate stone alone group (Group C), and healthy control group (Group D), with 25 people in each group.

### Inclusion criteria

Patients should meet the following criteria: at least 18 years old; diagnosed with upper urinary tract stone by urinary ultrasound, KUB, or CT; underwent surgical stone removal and stone composition analysis confirmed that the calcium oxalate content was ≥80%; MetS group met ≥3 of the following criteria (Peterseim et al., 2024): abdominal obesity (male waist circumference ≥90 cm, female waist circumference ≥85 cm), fasting blood glucose ≥6.1 mmol/L or a history of diabetes, blood pressure ≥130/85 mmHg or a history of hypertension treatment, triglycerides ≥1.7 mmol/L, male high-density lipoprotein cholesterol (HDL-C) < 1.04 mmol/L.

### Exclusion criteria

Exclusion criteria were as follows: pregnant women, patients with malignant tumors, patients with organic diseases of the digestive system (such as inflammatory bowel disease, irritable bowel syndrome, infections, tumors, or a history of bowel resection), patients who have used antibiotics or microecological agents in the last month, patients with secondary stone (due to anatomical anomalies of the urinary tract, metabolic disorders, or hereditary stone disease), and patients who have used calcium supplements, vitamins C or D, and metabolism-affecting drugs (such as corticosteroids, acetazolamide, etc.) for a long time.

### Clinical data collection

Using our hospital's electronic medical record system, the following data were collected from the subjects: gender, age, ethnicity, height, weight, body mass index (BMI), waist circumference, history of diagnosed diabetes mellitus and fasting blood glucose, history of diagnosed hypertension, triglycerides (TG), high-density lipoprotein cholesterol (HDL-C), low-density lipoprotein cholesterol (LDL-C), total cholesterol, serum total protein, albumin, serum sodium, potassium, calcium, magnesium, blood creatinine, urea nitrogen, uric acid, urine specific gravity, urine pH, urine leukocyte, urine protein, urine glucose, urine vitamin C, urine crystals, and stone composition analysis results.

### Specimen collection and preservation

Subjects took an appropriate amount of fecal specimen (5 ml) in a sterile collection tube using a sterile fecal sampler. All samples were collected at the beginning of the admission period (before antibiotic administration) and aseptic handling was used throughout. The specimens were stored in a refrigerator at −80°C immediately after collection.

### Specimen processing and bioinformatics analysis

Frozen samples were thawed and 0.2–0.5 g of fecal sample was taken and mechanically lysed using a Tissuelyser-48 Tissue Mill (60 Hz). Genomic DNA was extracted using OMEGA Soil DNA Kit, 0.8% agarose electrophoresis was used to assess integrity, and Nanodrop NC2000 was used to detect purity. Then the V3-V4 region (338F/806R primer) was amplified using high-fidelity enzyme (NEB Q5). Reaction program: pre-denaturation at 98 °C for 5 min; 25 cycles (98 °C for 30 s/53 °C for 30 s/72 °C for 45 s); final extension at 72 °C for 5 min. Products were verified by 2% electrophoresis and then purified by magnetic beads. After quantification and standardization, the library was constructed by TruSeq kit: end repair → addition of A-tail → junction ligation → magnetic bead screening → PCR enrichment. Single-stranded templates were prepared by NaOH denaturation after passing Agilent 2100 QC. Bipartite sequencing was carried out on MiSeq/NovaSeq platforms (300/500 cycles), and the recommended length of the target fragment was controlled in the 200–450 bp range. The target fragment length was recommended to be in the range of 200–450 bp. Raw data were preprocessed by QIIME2 (Bolyen et al., 2019) and OTUs were clustered using DADA2 (Callahan et al., 2016). Alpha diversity was assessed based on OTU distribution using Chao1 index, Shannon index, and Simpson index. Analysis of dimensionality was performed with PCoA and NMDS downscaling, ANOSIM analysis of inter- (intra-) group differences, and LEfSe multivariate statistical analysis of species differing between groups. LEfSe analysis (Segata et al., 2011) was applied to identify between-group differing species and attempts were made to find marker species. Redundancy analysis (RDA) methods were used to analyze potential associations between gut microbiota and relevant clinical parameters. KEGG metabolic pathway annotation based on PICRUSt2 (Douglas et al., 2020) algorithm to predict the metabolic function of gut microbiota and identify differential functional pathways, and use RStudio (Huber et al., 2015) and Python (Cock et al., 2009) to Draw statistical figures.

### Statistical methods

For the description of baseline characteristics, the median (quartiles) was used for continuous variables, while categorical variables were expressed as frequencies and percentages. For comparisons of continuous variables, the Student's *t*-test was used if the data conformed to a normal distribution, and the Mann-Whitney U-test was used if the data did not conform to a normal distribution. For comparisons of categorical variables, the χ^2^ test was used. All statistical analyses were performed using SPSS software version 26.0 and R language version 4.3.2, with a two-tailed *P*-value < 0.05 set as the threshold for statistical significance.

## Result

### Clinical characteristics of the four groups

There were no statistically significant differences among the four groups in terms of gender, age, and ethnicity (*P* > 0.05). The weights, BMIs, waist circumferences, prevalences of diabetes mellitus, hypertension, hyperlipidemia, and coronary heart disease in Groups A and B were significantly higher than those in Groups C and D (*P* < 0.05). The mean value of total serum protein in Groups A and B was significantly lower than that in Groups C and D (*P* < 0.05). The serum uric acid level (369.97 μmol/L) in Groups A and B was significantly lower than that in Groups C and D (*P* < 0.05). The serum uric acid levels differed significantly among the four groups (*P* = 0.003), with a higher level in Group A. Regarding the routine urine examination, the urine pH value in Group D was 6 (6.0–6.25), which was higher than that in the other groups, and the urine specific gravity was 1.016 (1.012–1.019), which was lower than that in the other groups (*P* < 0.05). The numbers of positive urine leukocytes and erythrocytes in Groups A and C were higher than those in Groups B and D (*P* < 0.05). The proportion of urinary crystals (69.2%) in Group A was significantly higher than that in Groups B and D (*P* = 0.001) (Table 1).

**Table 1:** Baseline table for the population of subjects in the four groups.

**Variables**	**Group-A (*n =* 25)**	**Group-B (*n =* 25)**	**Group-C (*n =* 25)**	**Group-D (*n =* 25)**	***P*-value**
Female (*n* %)	6 (26.1%)	5 (21.7%)	6 (26.1%)	6 (26.1%)	0.983
Age (years)	53.16 ± 11.08	51.48 ± 11.19	47.68 ± 13.00	53.60 ± 5.94	0.363
Ethnic (*n* %)	9 (23.7%)	9 (23.7%)	10 (26.3%)	10 (26.3%)	0.982
BMI (kg/m^2^)	28.84 ± 4.26	29.43 ± 3.0.67	24.37 ± 2.35	24.13 ± 1.81	< 0.001
WC (cm)	95.84 ± 11.35	103.12 ± 8.82	83.28 ± 4.37	81.32 ± 4.0.16	< 0.001
Hypertension (*n* %)	18 (47.37%)	20 (52.63%)	0 (0%)	0 (0%)	< 0.001
Diabetes (*n* %)	12 (38.7%)	19 (61.3%)	0 (0%)	0 (0%)	< 0.001
CAD (*n* %)	2 (22.2%)	7 (77.8%)	0 (0%)	0 (0%)	0.001
TG (mmol/L)	2.17 (1.74–3.11)	1.97 (1.15–2.44)	1.24 (0.91–1.46)	1.12 (0.89–1.50)	< 0.001
HDL – C (mmol/L)	0.86 (0.76–0.98)	0.87 (0.81–0.95)	1.2 (1.12–1.52)	1.19 (1.09–1.28)	< 0.001
TC (mmol/L)	3.77 (2.66–4.97)	4.43 (3.41–5.27)	4.26 (3.91–4.79)	4.82 (4.20–5.03)	0.138
LDL – C (mmol/L)	2.07 (1.38–3.16)	2.82 (2.11–3.55)	2.83 (2.47–3.20)	1.90 (1.21–2.25)	< 0.001
Total protein (g/L)	69.34 (66.03–72.50)	68.40 (63.96–72.55)	72.99 (67.30–67.30)	72.51 (70.09–73.69)	0.013
Albumin (g/L)	41.43 (38.76–43.42)	41.30 (38.63–43.20)	43.14 (40.04–46.30)	42.88 (40.84–44.08)	0.057
Glucose (mmol/L)	5.39 (4.68–7.09)	5.71 (4.98–8.23)	4.61 (4.36–4.91)	4.48 (4.45–4.54)	< 0.001
Sodium (mmol/L)	141.37 (140.13–143.09)	140.90 (138.50–143.02)	141.47 (140.02–143.04)	140.70 (139.45–142.00)	0.386
Potassium (mmol/L)	4.02 (3.80–4.23)	4.18 (3.94–4.26)	4.07 (3.69–4.27)	4.05 (3.70–4.29)	0.656
Calcium (mmol/L)	2.24 (2.14–2.30)	2.21 (2.15–2.28)	2.22 (2.17–2.33)	2.25 (2.12–2.45)	0.692
Magnesium (mmol/L)	0.87 (0.82–0.94)	0.87 (0.81–0.91)	0.91 (0.84–0.95)	0.82 (0.78–0.90)	0.097
Creatinine (μmol/L)	80.0.03 (68.30–107.00)	72 (62.4–89.0)	77.4 (59.5–92.3)	68 (53.55–77.5)	0.097
Urea nitrogen (mmol/L)	5.85 (5.00–7.22)	5.85 (5.43–7.60)	5.26 (4.51–7.24)	6.98 (5.067–7.31)	0.186
Uric acid (μmol/L)	369.97 (333.50–504.50)	319 (258.00–402.85)	335.5 (277.34–365.50)	314.51 (277.35–325.50)	0.003
Urine specific gravity	1.02 (1.01–1.03)	1.03 (1.02–1.03)	1.02 (1.01–1.02)	1.02 (1.01–1.02)	0.001
Urine pH	5.5 (5.0–5.75)	5.5 (5.0–6.0)	5.5 (5.25–6.0)	6.0 (6.0–6.25)	< 0.001
Urine leukocytes (*n* %)	11 (39.2%)	2 (7.2%)	14 (50%)	1 (3.6%)	< 0.001
Urine protein (*n* %)	16 (43.3%)	9 (24.3%)	9 (24.3%)	3 (8.1%)	0.002
Urine glucose (*n* %)	6 (35.3%)	11 (6.7%)	0	0	< 0.001
Urine vitamin C (*n* %)	0	0	0	0	1
Urine crystals (*n* %)	9 (69.2%)	2 (15.4%)	2 (15.4%)	0	0.001

### Differences in bacterial diversity between groups

**Figure 1:**
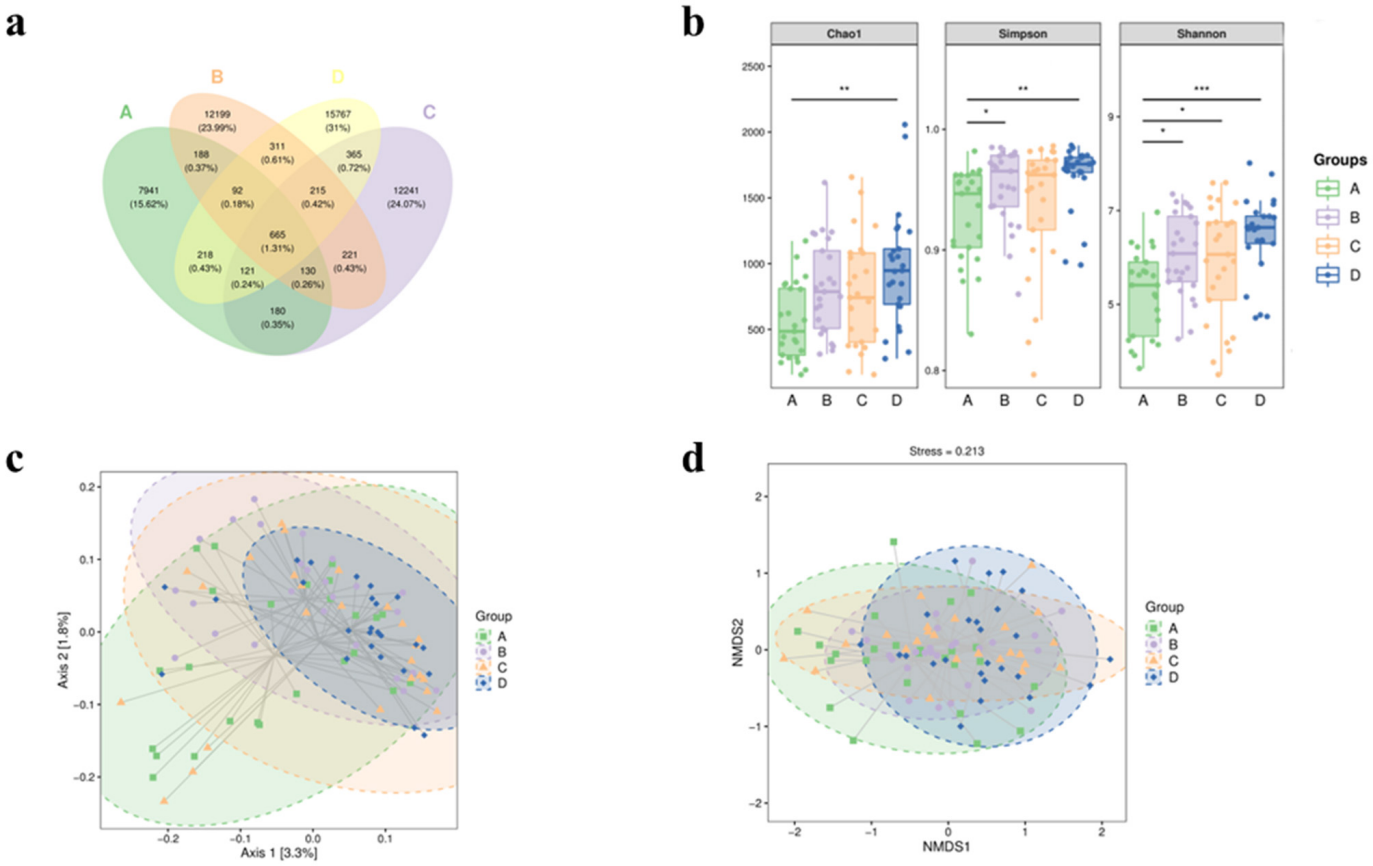
The α diversity and β diversity indices of fecal intestinal microbiota in four groups of patients. **(a)** Venn plot of OTU. **(b)** Block diagram of grouping based on Chao1, Shannon and Simpson's α diversity indices. Bacterial β-diversity map based on Jaccard distance **(c)** PCoA; **(d)** NMDS. A represents the group of calcium oxalate stone combined with metabolic syndrome, B the metabolic syndrome alone group, C the calcium oxalate stone alone group, and D the healthy control group. *p < 0.05, **p < 0.01, ***p < 0.001.

The 16S rRNA sequencing data were clustered into OTUs (operational taxonomic units) according to 97% similarity, and the Venn diagrams showed the unique and shared status of each group: A (*n* = 7,941), B (*n* = 12,199), C (*n* = 12,241), and D (*n* = 15,767), the number of overlapping OTUs among the four groups is (*n* = 665) (Figure 1a).

In terms of Chao1 index, which reflects species richness, statistically significant differences were found between Groups A and D (*P* = 0.0017), while there were no significant differences among the other groups. The Shannon index reflecting diversity was found to be significantly different between Groups A and B (*P* = 0.04), A and C (*P* = 0.048), and A and D (*P* = 0.00027). On the contrary, no significant differences were observed in the comparison between the other groups. Similarly, Simpson's index showed significant differences between Group A and Group B (*P* = 0.04) and Group A and Group D (*P* = 0.0016), but not between Group A and Group C (*P* = 0.24). Comparisons between the other groups remained insignificant (Figure 1b). β-diversity analysis was performed using the Jaccard distance matrix, visualized by PCoA and NMDS downscaling (Figure 1c), which showed that the composition of the microbiota in Group A was significantly different from that of the other groups. The ANOSIM test confirmed the differences between the groups (R > 0, *P* < 0.05) (Table 2).

**Table 2:** Results of ANOSIM analysis between groups.

**Groups**	**R-value**	***P*-value**
A-B	0.186981	0.001
A-C	0.131565	0.002
A-D	0.241469	0.001
B-C	0.141365	0.001
B-D	0.182568	0.001
C-D	0.118344	0.002

### Analysis of species differences

**Figure 2:**
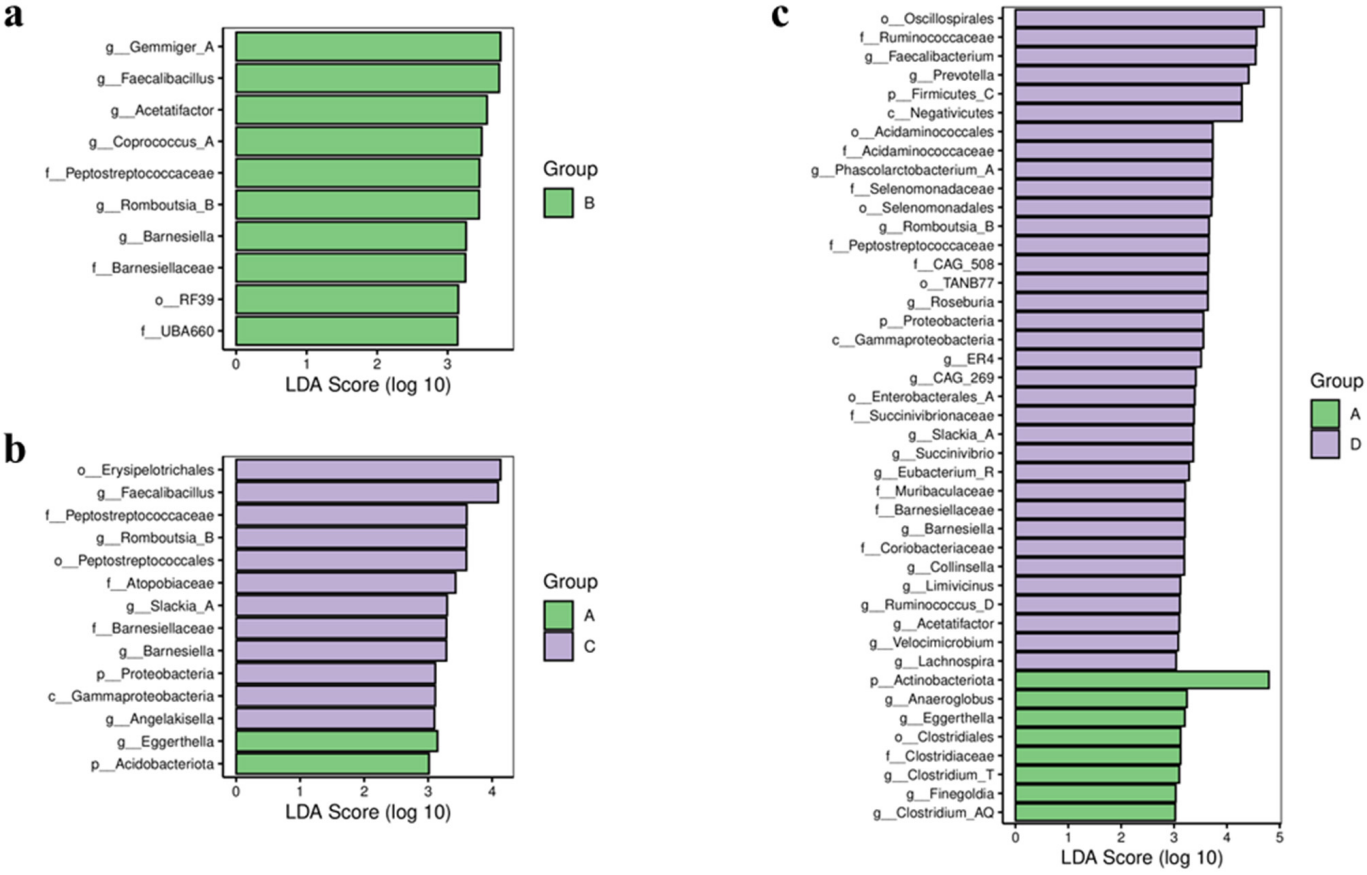
Bar chart of LDA effect sizes for differential species between groups. LDA scores (log 10) >3 and *p* < 0.05 are listed. **(a)** Group A vs. group B. **(b)** Group A vs. group C. **(c)** Group A vs. group D. A represents the group of calcium oxalate stone combined with metabolic syndrome, B the metabolic syndrome alone group, C the calcium oxalate stone alone group, and D the healthy control group.

Species differences were analyzed by the LEfSe method with the LDA effect size threshold set at 3.0. Compared to Group A, Group B had a higher abundance of taxa primarily at the family *Peptostreptococcaceae, Barnesiellaceae*, etc., and genus *Gemmiger_A, Faecalibacillus*, etc. (Figure 2a).

Compared to Group A, Group C showed distinct taxonomic profiles. Group A was enriched in the *Acidobacteriota* phylum and *Eggerthella* genus. In contrast, Group C had higher relative abundances of the *Proteobacteria* phylum; *Gammaproteobacteria* class; the order *Peptostreptococcales* and *Erysipelotrichales*; family including *Peptostreptococcaceae* and *Atopobiaceae*, etc.; and genus such as *Romboutsia_B, Barnesiella, Angelakisella*, etc. (Figure 2b).

The comparison between Group A and Group D further revealed significant differences. Group A was characterized by a higher abundance of the *Actinobacteria* phylum, the order *Clostridiales*, and genus including *Clostridium_T, Eggerthella, Anaerococcus*, etc. Conversely, Group D was enriched across multiple taxonomic levels, comprising the *Firmicutes_C* and *Proteobacteria phyla*, among others; the *Negativicutes* and *Gammaproteobacteria* classes, etc.; the order *Selenomonadales, Oscillospirales, Acidaminococcales*, etc.; family such as *Ruminococcaceae, Veillonellaceae, Selenomonadaceae*, etc.; and genus including *Faecalibacterium, Prevotella, Roseburia*, etc. (Figure 2c).

### Correlation analysis of microbiota alterations with clinical parameters

Redundancy Analysis (RDA) was used to examine the relationship between microbial communities and clinical parameters by constrained ordination. At the genus level, we screened the top 30 dominant genera in terms of relative abundance in all fecal samples for correlation analysis with the collected clinical data and plotted correlation heatmaps.

**Figure 3:**
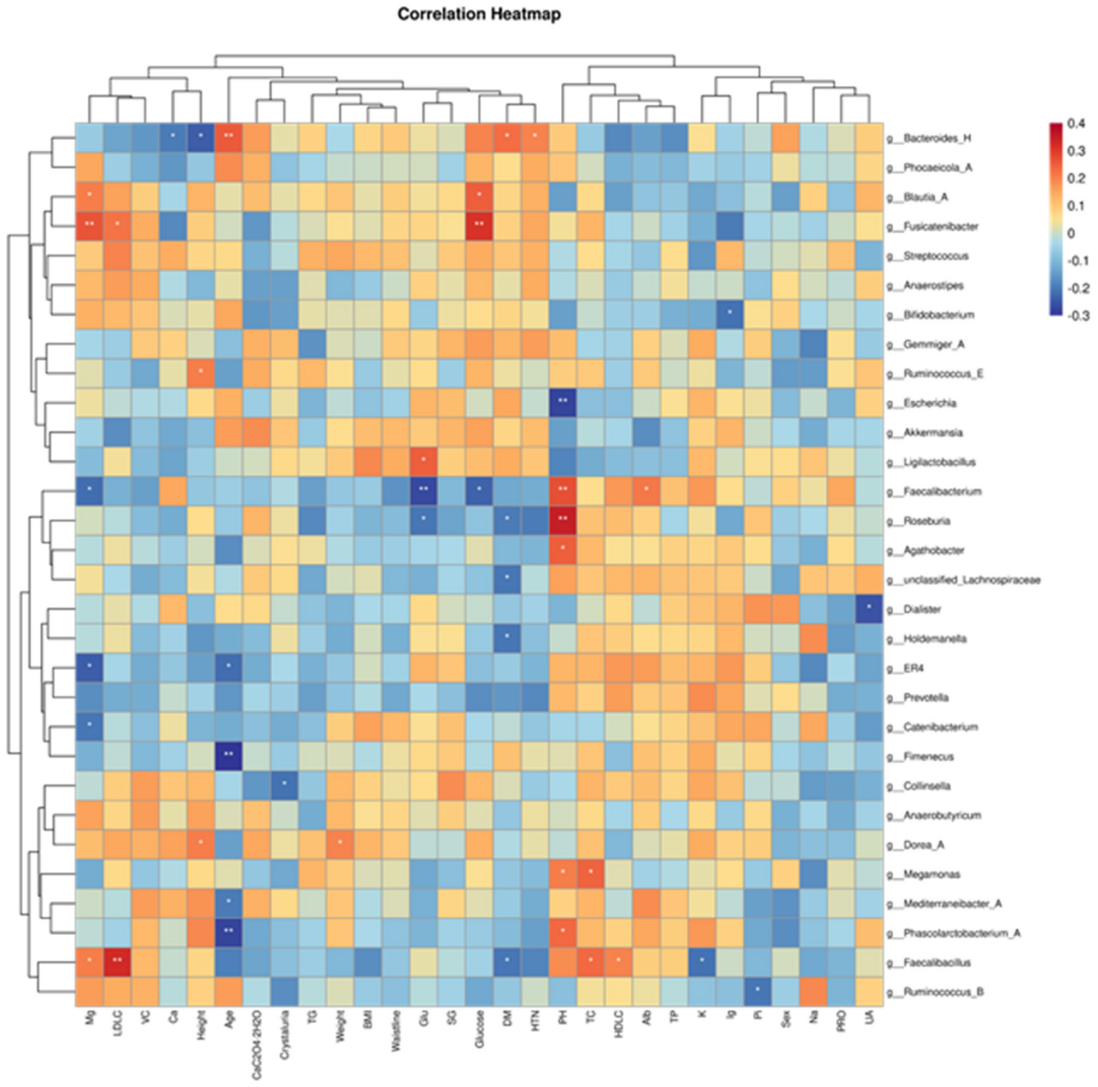
Heatmaps of correlations between gut microbiota abundance and relevant clinical characteristics. The horizontal coordinates are the clinical parameters and the vertical coordinates are the 30 most abundant genera.

The results showed that *Fusicatenibacter* and *Blautia-A* are positively correlated with blood glucose, while *Faecalibacterium* is negatively correlated with blood glucose. *Faecalibacillus* is positively correlated with low-density lipoprotein and high-density lipoprotein. *Bacteroides-H* is negatively correlated with blood calcium ions and positively correlated with hypertension. *Roseburia* and *Faecalibacterium* are positively correlated with urine pH, while *Escherichia* is negatively correlated with urine pH. *Collinsella* is negatively correlated with urinary crystals. The genus *Fusicatenibacter* is positively correlated with magnesium ions and negatively correlated with urinary glucose (Figure 3).

### Functional prediction of the gut microbiota based on the KEGG database

After obtaining the abundance data of metabolic pathways through KEGG functional annotation, we employed metagenomeSeq analysis to identify the functional pathways that were significantly different between groups. Compared with Group B, the retinol metabolism pathway is enriched in Group A, while the bisphenol degradation pathway is reduced. Compared with Group C, the photosynthesis - related pathways are enriched in Group A, and the toxoplasmosis-related pathways are reduced. Compared with Group D, the beta-alanine metabolism and photosynthesis pathways are enriched in Group A (Table 3).

**Table 3:** Results of differential analysis of KEGG signaling pathway.

**Group**	**Pathway**	**logFC**	**se**	***P* values**
A-B	Retinol_metabolism	0.9284	0.3355	0.00566
	Bisphenol_degradation	−1.45	0.6569	0.02729
A-C	Photosynthesis	0.9691	0.4463	0.02988
	Toxoplasmosis	−0.2659	0.133	0.04562
A-D	Beta-Alanine_metabolism	0.4742	0.1824	0.00933
	Photosynthesis	0.9604	0.4703	0.04116

## Discussion

The formation of primary calcium oxalate stone is influenced by a variety of factors, including genes, metabolism, diet, lifestyle, and the environment, and there is a lack of an exact mechanism to explain the formation process (Shastri et al., 2023). Therefore, there are currently no effective preventive or pharmacological measures against the formation and recurrence of calcium oxalate stone. Several studies have shown that metabolic abnormalities play an important role in the formation of calcium oxalate stone, and metabolic syndrome, as a collection of metabolic abnormalities in human metabolism, is considered to be an important risk factor for the formation and recurrence of calcium oxalate stone (Kohjimoto et al., 2013). The gut, a critical metabolic organ, is predominantly regulated by the intestinal microbiota. Recent studies have established robust associations between gut microbiota composition and both metabolic syndrome and nephrolithiasis (Ezenabor et al., 2024; Hussain et al., 2024). While metabolic syndrome may enhance calcium oxalate stone formation via the gut microbiota, direct evidence is scarce. To address this, our study profiles the gut microbiota of patients who have both calcium oxalate stones and metabolic syndrome.

Our analysis revealed distinct gut microbiota characteristics in patients with calcium oxalate stone comorbid with metabolic syndrome (CaOx-MetS). Specifically, the CaOx-MetS group exhibited significantly lower α diversity compared to the metabolic syndrome-only (MetS-only), stone-only, and healthy control groups. β diversity analysis further demonstrated a marked divergence in microbial community structure between the CaOx-MetS group and the other three cohorts. This reduction in microbial diversity was accompanied by a diminished abundance of beneficial bacteria, which may compromise critical metabolic functions of the gut microbiota. Such dysbiosis could impair systemic resistance to pathogenic stimuli, thereby elevating the overall risk of disease pathogenesis (Pickard et al., 2017). In the context of nephrolithiasis, impaired metabolic functions—particularly in oxalate degradation and short-chain fatty acid (SCFA) production—may directly exacerbate stone formation. SCFAs exert anti-lithogenic effects through multiple mechanisms, such as modulating intestinal pH, inhibiting oxalate absorption, and alleviating inflammatory responses (Liu et al., 2021).

LEfSe analysis revealed significant reductions in the relative abundances of *Gemmiger_A, Faecalibacillus, Acetatifactor*, and *Coprococcus_A* in the calcium oxalate stone with metabolic syndrome (CaOx-MetS) group compared to the metabolic syndrome-only group. Duan et al. (2021) observed that obese patients exhibit decreased gut microbiota diversity, with the genus *Gemmiger* showing significantly lower abundance in obese individuals than in healthy controls. *Faecalibacillus* is a genus of exclusively anaerobic bacteria, and less research has been done on *Faecalibacillus*. *Faecalibacillus* has good probiotic potential, producing SCFA, such as butyric acid, through fermentation of dietary fibers, a metabolite that is essential for maintaining intestinal barrier integrity, modulating immunity, and suppressing inflammation. Experiments in mice have found that *Faecalibacillus* isolates provide better relief from constipation (Huang et al., 2024). Another study found a relative decrease in the abundance of *Faecalibacillus* in a variety of malignant tumors such as gastrointestinal tumors, lung cancer, and breast cancer (Kulecka et al., 2024). The genus *Acetatifactor* exhibits bile salt hydrolase (BSH) activity, converting deoxycholic acid to lithocholic acid. This metabolite activates the TGR5 signaling pathway, stimulating intestinal L-cells to secrete glucagon-like peptide-1 (GLP-1), which promotes adipose tissue browning and enhances hepatic insulin signaling and glucose metabolism (Pathak et al., 2018). Additionally, *Coprococcus_A* generates butyrate, a key SCFA member essential for maintaining gut homeostasis, regulating inflammation, and reinforcing intestinal barrier integrity (Wang et al., 2024). It can be observed that in patients with calcium oxalate stone complicated by metabolic syndrome, the abundance of probiotics is reduced. These diminished probiotics exacerbate metabolic disorders, thereby promoting the development of kidney stone in patients with metabolic syndrome.

Compared to the stone-only group, the calcium oxalate stone with metabolic syndrome (CaOx-MetS) group exhibited significant enrichment of *Acidobacteriota* and *Eggerthella*. In contrast, the stone-only group demonstrated higher abundances of *Erysipelotrichales, Faecalibacillus*, and *Peptostreptococcaceae*. To date, no definitive evidence links Acidobacteriota directly to oxalate or short-chain fatty acid (SCFA) metabolism or urolithiasis. However, studies have reported elevated *Acidobacteriota* abundance in patients with neurodegenerative disorders such as Alzheimer's disease and amyotrophic lateral sclerosis (ALS). This enrichment of potentially pathogenic taxa may impair intestinal barrier integrity and trigger pro-inflammatory responses, thereby contributing to disease progression (Fontdevila et al., 2024; Heravi et al., 2023). *Eggerthella*, a Gram-positive bacillus, is frequently overrepresented in conditions including gastrointestinal inflammation and neurological diseases (Nikolova et al., 2021). Its pathogenic potential may arise from mechanisms such as increased intestinal permeability and systemic oxidative stress, which could indirectly exacerbate both metabolic syndrome and stone formation. Notably, *Peptostreptococcaceae*, a SCFA-producing family, has been associated with reduced diabetes risk and confers protective effects against metabolic syndrome (Chen et al., 2021). The observed reduction in SCFA-producing taxa (e.g., *Faecalibacillus, Peptostreptococcaceae*) in the CaOx-MetS group compared to the stone-only group underscores a dual depletion of beneficial microbiota and exacerbated microbial dysbiosis in comorbid patients.

When compared with healthy controls, the calcium oxalate stone combined with MetS group exhibited a greater enrichment of harmful bacteria and a decrease in health-related microbiota. Specifically, the abundance of *Anaeroglobus, Eggerthella*, and *Finegoldia* increased, whereas that of *Faecalibacterium, Prevotella, Roseburia*, and *Acetatifactor* relatively decreased. Previous studies indicate that *Faecalibacterium* abundance is markedly decreased in stone patients and is inversely correlated with stone formation, likely via its roles in oxalate metabolism and SCFA production (Ticinesi et al., 2018). Meta-analysis showed that *Prevotella* was significantly less abundant in patients with stone than in the healthy population, important protective bacteria (Yuan et al., 2023). In addition *Roseburia* and *Acetatifactor*, both SCFA-producing bacteria, calcium oxalate stone have a protective effect on the gut microbiota (Liu et al., 2022).

In this study, *Fusicatenaibacter* and *Blautia_A* abundance was negatively correlated with blood glucose levels, *Faecalibacterium* and *Roseburia* abundance was positively correlated with urinary PH levels, in addition, *Collinsella* was negatively correlated with urinary crystallization. It can be seen that the dysbiosis occurring in the group of calcium oxalate stone combined with metabolic syndrome may contribute to the development of calcium oxalate stone by affecting the metabolism and the physicochemical properties of the urine of the patients and, consequently, to the development of calcium oxalate stone. The exact mechanism needs to be confirmed by further research.

Furthermore, comparative analysis of metabolic pathway activity across groups revealed that the calcium oxalate stone with metabolic syndrome (CaOx-MetS) group exhibited significantly reduced bisphenol degradation pathway activity compared to the metabolic syndrome-only group (*P* = 0.0273), alongside elevated retinol metabolism pathway activity (*P* = 0.0057). Bisphenol A (BPA), a prevalent industrial chemical, is extensively utilized in the production of polycarbonate plastics and epoxy resins. As an endocrine-disrupting compound, BPA interferes with multisystem physiological functions, triggering diverse pathological effects. Its lipophilic nature facilitates accumulation in adipose tissue, where it disrupts adipokine secretion and exacerbates obesity and insulin resistance. Additionally, BPA induces cellular apoptosis via caspase-dependent mitochondrial damage pathways and impairs insulin signaling by modulating ion channel activity, particularly potassium channels (Akash et al., 2020). Enhanced bisphenol degradation capacity is hypothesized to mitigate BPA-driven metabolic dysregulation. Critically, BPA exposure may provoke renal tubular injury, impairing reabsorption and secretory functions. BPA-induced oxidative stress further damages tubular epithelial cells, amplifying lithogenic susceptibility (Jacobson et al., 2020). The progressive decline in bisphenol degradation capacity observed in comorbid patients suggests heightened sensitivity to environmental xenobiotics. Retinol (vitamin A), a fat-soluble vitamin essential for human health, is absorbed in the small intestine and metabolized in the liver to yield retinoic acid (RA), a pivotal signaling molecule (Rastinejad, 2022). All-trans retinoic acid (ATRA), a specific RA isomer, serves as a renal protective factor. It attenuates inflammatory cytokine expression, protects proximal tubular epithelial cells from hypoxic injury, and promotes tubular epithelial differentiation, thereby exerting renoprotective and reparative effects in chronic kidney disease (Nakamura et al., 2019). Our findings align with a pediatric nephrolithiasis metabolomics study (Wen et al., 2021) that identified upregulated retinol metabolism as a compensatory mechanism against crystal-induced tubular damage. Collectively, these results suggest that the depletion of protective metabolic pathways (e.g., bisphenol degradation) and compensatory activation of retinol metabolism may synergistically contribute to the heightened risk of stone formation in metabolic syndrome patients.

The following limitations exist in this study: The sample size of this study is limited. The cross-sectional design makes it difficult to establish a causal relationship, and longitudinal cohort studies, probiotic interventions, and fecal microbial transplantation (FMT) animal experiments are needed to verify the clinical value of the differential microbiota described above. The difference between the numbers of diabetes and obesity in the calcium oxalate stone combined with metabolic syndrome group and the metabolic syndrome group in this study may lead to bias, and further studies need to be conducted in the later stage by matching each metabolic syndrome composition one to one and expanding the sample size. Future studies could combine gut microbiota and urinary microbiota to analyze alterations in the microbiome at multiple sites. Gut microbiota analysis combined with metabolomics will allow in-depth analysis of the molecular mechanisms of key strains and explore therapeutic options such as probiotics, or microbiota transplantation.

## Conclusion

This study reveals a “dual imbalance” in the gut microbiota of patients with calcium oxalate stone combined with metabolic syndrome, characterized by an enrichment of pro-inflammatory bacteria and a relative decrease in SCFA-producing bacteria. This dysbiosis may contribute to metabolic abnormalities by exacerbating oxidative stress and inflammation, which may contribute to stone formation. These findings provide a rationale for the development of interventional strategies targeted at the microbiota.

## Data Availability

The data presented in this study are publicly available. This data can be found here: https://www.ncbi.nlm.nih.gov/sra, accession PRJNA1273845.
